# A Critical Review of the Biochemical Mechanisms and Epigenetic Modifications in HIV- and Antiretroviral-Induced Metabolic Syndrome

**DOI:** 10.3390/ijms222112020

**Published:** 2021-11-06

**Authors:** Jivanka Mohan, Terisha Ghazi, Anil A. Chuturgoon

**Affiliations:** Discipline of Medical Biochemistry, School of Laboratory Medicine and Medical Sciences, University of KwaZulu-Natal, Durban 4041, South Africa; mxtjivee@gmail.com (J.M.); terishaghazi@gmail.com (T.G.)

**Keywords:** metabolic syndrome, HIV, ARVs, mitochondrial dysfunction, inflammation, epigenetics

## Abstract

Metabolic syndrome (MetS) is a non-communicable disease characterised by a cluster of metabolic irregularities. Alarmingly, the prevalence of MetS in people living with Human Immunodeficiency Virus (HIV) and antiretroviral (ARV) usage is increasing rapidly. This study aimed to look at biochemical mechanisms and epigenetic modifications associated with HIV, ARVs, and MetS. More specifically, emphasis was placed on mitochondrial dysfunction, insulin resistance, inflammation, lipodystrophy, and dyslipidaemia. We found that mitochondrial dysfunction was the most common mechanism that induced metabolic complications. Our findings suggest that protease inhibitors (PIs) are more commonly implicated in MetS-related effects than other classes of ARVs. Furthermore, we highlight epigenetic studies linking HIV and ARV usage to MetS and stress the need for more studies, as the current literature remains limited despite the advancement in and popularity of epigenetics.

## 1. Introduction

The World Health Organisation (WHO) describes MetS as a pathological condition characterised by a cluster of metabolic irregularities, including abdominal obesity, insulin resistance, atherogenic dyslipidaemia, and hypertension [1]. The syndrome is diagnosed by having at least three of the following symptoms: central obesity, elevated triglycerides, reduced high-density lipoprotein cholesterol levels, high blood pressure, and elevated fasting glucose levels [2,3,4].

MetS is considered a global health hazard, with an estimated 20–30% of adults being affected [5]. It contributes to the development of other pathological conditions such as Type 2 Diabetes Mellitus (T2DM), cardiovascular diseases (CVD), and hypertension [1]. Therefore, MetS has been implicated in widespread morbidity and mortality [5].

The biochemistry of MetS has been linked to chronic systemic inflammation and oxidative stress [6,7]. Consequently, the literature concludes that the disease is strongly associated with processes that contribute to the excess production of reactive oxygen species (ROS) and cytokine activation, such as mitochondrial dysfunction and inflammatory pathways [5]. More recent advancements in science have highlighted the role of epigenetics in MetS progression. This field of study is novel and remains elusive; however, promising studies are emerging with data that provide mechanistic insights into epigenetic modifications and MetS occurrence [8].

Interestingly, there is an increase in the occurrence of MetS in HIV-infected individuals [9]. HIV affects a significant percentage of the global population. By the end of 2019, roughly 38 million people worldwide lived with HIV, with 1.7 million new infections for the year [10]. In 2020, the South African government reported that 7.8 million people were infected with HIV [11]. The high prevalence of HIV, especially in South Africa, emphasises the need for studies on co-morbidities such as MetS. Research on the subject may provide insight for possible treatments to reduce the prevalence of MetS associated with HIV.

Furthermore, 26 million HIV-infected individuals had access to ARV treatment at the end of 2019 [10]. The use of highly active antiretroviral therapy (HAART) in HIV-infected patients has decreased the HIV-infected population’s mortality, with 15.3 million lives saved in 2019 [12]. However, the use of HAART promotes metabolic complications that resemble MetS in people living with HIV (PLWH) [13,14]. The prevalence of MetS following ARV treatment varies over different studies, with some exceeding 30%, further highlighting the need for future studies [15].

Considering the high incidence of HIV and MetS, it is vital to understand the relationship between the two conditions and the use of antiretroviral drugs (ARVs). This study aimed to explore mechanisms used by (a) the HIV infection and (b) different classes of HAART to induce metabolic irregularities. This review establishes the biochemical mechanisms of HIV and ARVs in MetS-related problems such as mitochondrial dysfunction, insulin resistance, inflammation, dyslipidaemia, and lipodystrophy. Furthermore, we look at epigenetic modifications induced by ARVs and HIV that result in metabolic complications. More specifically, the review highlights gaps in current knowledge and addresses the need for further studies to reduce inconsistencies in research.

## 2. HIV and MetS

Many risk factors contribute to the incidence of MetS, including an unhealthy diet, lack of exercise, and age [16]. However, research has established a unique set of risk factors associated with HIV infection [17]. The common risk factors highlighted in HIV infection include chronic inflammation and immune dysfunction, which promotes atherosclerosis, dyslipidaemia, and T2DM [18].

At least 690,000 people had died from HIV-related illness (including metabolic complications) by the end of 2019. The majority of the global HIV-infected population (20%) resides in South Africa [10]. In multiple South African community studies, it was determined that the prevalence of MetS ranges from 24.1 up to 60.6%, with mostly females being affected [19,20,21]. Furthermore, following four cross-sectional studies in sub-Saharan Africa, a prevalence of 21.5% MetS in PLWH was found, whereas un-infected individuals had a 12% prevalence [9]. Therefore, the incidence of MetS in HIV is significant enough to warrant extensive research.

**Figure 1:**
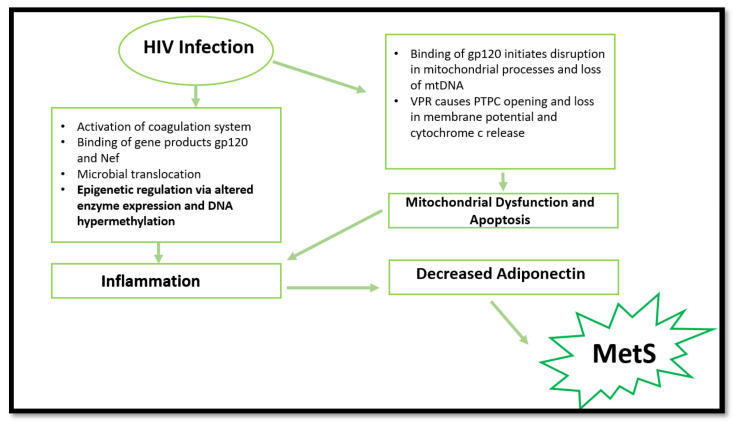
Summary of processes involved in MetS promotion via HIV infection. HIV infection can cause mitochondrial dysfunction, apoptosis, epigenetic changes, and inflammation, resulting in decreased adiponectin expression. Consequently, MetS initiation occurs. (gp120—envelope glycoprotein GP120; Nef—negative factor; DNA—deoxyribonucleotide acid; mtDNA—mitochondrial DNA; VPR—viral protein R; PTPC—permeability transition pore complex).

The biochemical basis of HIV-induced MetS remains elusive; however, research has established a few common factors. HIV can induce MetS via several mechanisms (Figure 1). The most common is via the activation of inflammatory responses, cellular apoptosis, and mitochondrial dysfunction. However, epigenetic modifications are emerging in recent research surrounding HIV and inflammation. The ability to induce the aforementioned pathways and changes leads to more severe consequences, such as insulin resistance, dyslipidaemia, and obesity [2].

We discuss the most common mechanisms used by HIV to induce MetS in PLWH.

## 3. HIV and Inflammation

Untreated HIV infection has been linked to the activation of the coagulation system, which results in the production of pro-inflammatory molecules, including cytokines and chemokines. Following treatment of HIV with HAART, inflammation persists through high levels of interleukin IL-6, C-reactive proteins, and D-dimers [22].

Early research shows that HIV gene products can directly trigger lymphocyte and macrophage activity and promote the production of pro-inflammatory cytokines and chemokines. The HIV envelope protein gp120 directly activates immune cells or increases their susceptibility to activation by binding to CD4 receptors and co-receptors [23,24,25]. Furthermore, the HIV accessory molecule Nef can activate lymphocytes or cause indirect activation via infection of macrophages [26,27,28].

The literature describes HIV-associated microbial translocation as a tool for persistent inflammation. HIV is linked to increased lipopolysaccharide (LPS) levels (an indicator of microbial translocation) [29]. Increased LPS concentrations stimulate macrophage and dendritic cells to produce inflammatory molecules, including tumour necrosis factor alpha (TNF-α), IL-6, and IL-1β. This establishes the pro-inflammatory state commonly related to the virus [30]. More specifically, inflammatory states are initiated via the activation of inflammasomes. Upon stimulation by LPS, Toll-like receptors signal for NLRP3 inflammasome activation. These protein complexes ultimately allow for the autoproteolytic activation of pro-caspase-1 to yield caspase-1. The latter is responsible for the conversion of pro-IL-1β to IL-1β, which creates inflammatory conditions [31]. The activation of inflammasomes in HIV infection encourages the occurrence of metabolic disorders [32].

Chronic pro-inflammatory environments promote conditions such as T2DM and CVD [33,34]. The increase in inflammation causes an inhibition in the function of adiponectin—a protein hormone involved in glucose regulation and fatty acid breakdown. Adiponectin is associated with anti-diabetic, anti-atherosclerotic, and anti-inflammatory functions that suppress MetS progression. Therefore, inhibition results in enhanced insulin resistance and atherosclerosis, further manifesting into T2DM and CVD, respectively [35].

Although there are multiple adipocytokines involved in the prevention of the pathogenesis of MetS, adiponectin had been the most commonly described in the literature. There are several possibilities for adiponectin to reduce insulin resistance; however, this review describes the most common mechanism. In summary, adiponectin binds to the appropriate receptor and activates various intracellular pathways. More frequently, it allows for the activation of the AMP-activated protein kinase (AMPK) pathway, which results in AMPK phosphorylation [36]. The latter promotes glucose utilization which increases fatty acid oxidation. Additionally, it encourages glucose uptake in muscle cells through increased glucose transport 4 (GLUT4) translocation and reduces gluconeogenesis in the liver [37,38]. AMPK is associated with increased insulin sensitivity and a reduction in glucotoxicity oxidative stress in target cells/organs [38]. Therefore, lower adiponectin concentrations or inhibition of its function promotes the occurrence of insulin resistance and fat accumulation that can lead to other clinical outcomes of MetS.

## 4. HIV, Mitochondrial Dysfunction, and Cell Apoptosis

Mitochondrial dysfunction is commonly observed in PLWH. As previously mentioned, the gp120 protein binds to the CD4 receptor and co-receptors to elicit infection. Such binding initiates pathogenic effects, including mitochondria-mediated apoptosis, loss of mitochondrial DNA (mtDNA), and impaired calcium signalling [39]. It is well understood that dysregulation of mitochondrial function results in the production of inflammatory cytokines, including TNF-α, interleukins, and C-reactive proteins [40].

In other instances, HIV may induce MetS by inducing mitochondrial membrane dysfunction [41]. Viral protein R (VPR), an HIV accessory molecule, can cause mitochondrial permeability transition pore complex (PTPC) opening and loss of transmitochondrial potential upon mitochondrial exposure [42]. The opening of the PTPC disrupts mitochondrial processes and releases proapoptotic factors such as cytochrome c and procaspase 9 [42,43]. HIV promotes the uncontrolled release of cytochrome c, thus increasing apoptosis. Consequently, a pro-inflammatory state is favoured. Inflammatory cytokines suppress adiponectin function and impair insulin function in muscles. This ultimately leads to MetS [40,44].

## 5. HIV and Epigenetic Modifications

Mechanisms surrounding epigenetic modifications remain elusive; however, some research has highlighted possible linkages. This provides motivation for further extensive research to be carried out to fully explain the processes involved in the area.

Aside from the common mechanisms, more recent research has suggested that HIV-1 infection can cause epigenetic changes when exposed to *Mycobacterium tuberculosis*. The latter results in altered monocyte function and dysregulation in pro-inflammatory cytokine production. The same study suggested that a decrease in global DNA methylation occurred in HIV-infected individuals. This was mainly attributed to the downregulation of DNA methyltransferases and the upregulation of methyl-CpG-binding proteins. Consequently, the reduction in global DNA methylation caused an increased activation status of monocytes. This result was accompanied by increased production of pro-inflammatory cytokines [45]. These findings are significant, considering the vulnerability to *Mycobacterium tuberculosis* in developing countries with high HIV prevalence, such as South Africa. The study provides plausible cause to initiate future in vivo research that will aid in understanding epigenetic changes associated with HIV and MetS.

More recent findings report DNA hypermethylation in HIV-infected patients. These epigenetic modifications result in cell dysfunction and decreased cytokine production, thus increasing CVD and T2DM risk [46]. More specifically, the HIV-1 infection causes epigenetic changes in the T-cell population, resulting in aberrant expression of pro-inflammatory cytokines and immune-related genes [47]. This is caused by the virus inducing DNA methylation changes in essential genes (IL-2, PD-1 and FOXP3) in T-cells which initiates cell dysfunction [48,49,50].

Furthermore, single-nucleotide polymorphisms in IL-6 and IL-10 were associated with accelerated ageing and the dysregulation of inflammatory responses in PLWH [46]. However, the lack of studies on related polymorphisms causes gaps in knowledge regarding the mechanisms.

The overall evidence strongly suggests that HIV can induce various epigenetic modifications that may lead to disarray in inflammatory responses. The sparse literature on HIV and epigenetics emphasises the necessity for more research on the subject, to further underline the virus’s role in MetS progression.

## 6. The Evolution of ARVs and Implications in MetS

The use of ARVs provides temporary relief for HIV-induced effects but has induced various complications over time (Masuku et al., 2019). Side effects associated with the use of ARVs have improved following extensive research. Previously, adverse outcomes seen with ARV usage resulted in patients discontinuing treatment or switching combinations of drugs until the side effects were manageable. This was mainly observed in first- and second-generation treatment (O’Brien et al., 2003). Initially, the discovery of ARVs led to various trials of singular and combinational usage, with NNRTIs and NRTIs being popular. However, the usage of the earlier generations of ARVs were later associated with adversity and HIV drug resistance (WHO, 2020). Fortunately, the newer generation of ARVs are linked with fewer complications and adversity (Barnhart and Shelton, 2015; Rai et al., 2018). More specifically, the World Health Organization has highlighted the need to move to newer generations of ARVs such as dolutegravir to prevent the HIV drug resistance associated with former generations and reduce side effects (WHO, 2020). However, some problems still arise following the usage of the current generation of ARVS.

**Figure 2:**
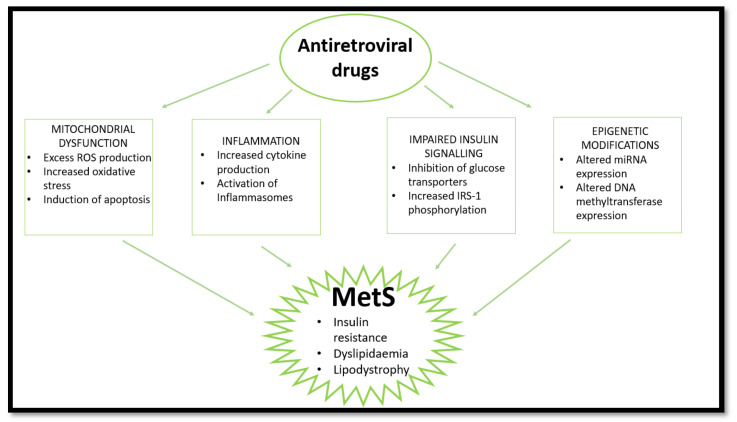
HAART induces MetS via several pathways. HAART causes mitochondrial dysfunction, inflammation, and impaired insulin signalling and epigenetic changes, leading to insulin resistance, lipodystrophy, and dyslipidaemia. (ROS—reactive oxygen species; IRS-1—insulin receptor substrate 1; miRNA—micro ribonucleotide acid).

Long-term use of ARVs is associated with the development of MetS through induction of dyslipidaemia, lipodystrophy, mitochondrial dysfunction, and insulin resistance [51]. Over time, metabolic dysregulation occurs, initiating changes in fat distribution and glucose homeostasis [52]. The consensus in population studies indicates a high prevalence of MetS in PLWH receiving ARV treatment [9,53,54]. This review looks at the effects of nucleoside reverse transcriptase inhibitors (NRTIs), non-nucleoside reverse transcriptase inhibitors (NNRTIs), protease inhibitors (PIs), and integrase strand transfer inhibitors (INSTIs) on mitochondrial dysfunction, insulin resistance, inflammation, and lipodystrophy/dyslipidaemia which are common markers and outcomes of MetS (Figure 2). Currently, the literature indicates that PIs are most commonly implicated in MetS cases. 

The role of the mitochondria in the pathogenesis of MetS is not fully understood. However, it is well noted that mitochondrial dysfunction contributes to inflammation, ROS production, and oxidative stress, which is strongly associated with MetS [5]. More specifically, mitochondrial dysfunction and inflammation are considered to be the underlying processes that lead to insulin resistance. In other instances, it was found that a side effect of ARV usage was the induction of dyslipidaemia and lipodystrophy—a clinical outcome of MetS. Below, we look at the different classes of ARVs and their role in the initiation or promotion of MetS.

## 7. Non-Nucleoside Reverse Transcriptase

NNRTIs were the first ARVs to receive regulatory approval for treatment of HIV. The class consists of drugs that have diverse drug backgrounds but similar mechanisms of action. They work by binding to the HIV-1 reverse transcriptase and inducing conformational changes that reduce the function of the enzyme [55]. However, NNRTIs were found to be problematic in several studies.

**Figure 3:**
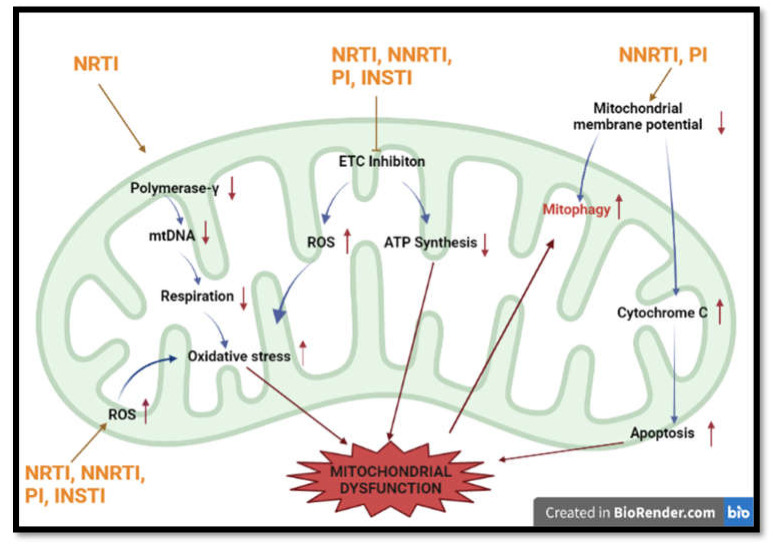
Summary of most common methods implicated in HAART-induced mitochondrial dysfunction. HAART induces mitochondrial toxicity via inhibition of respiration, production of ROS, induction of apoptosis and interference with mtDNA numbers. (Polymerase-γ —Polymerase gamma; ETC—electron transport chain; ATP— adenosine triphosphate; mtDNA-mitochondrial DNA).

The most common NNRTI is efavirenz. Efavirenz is associated with increases in apoptosis, increased mitochondrial mass, and oxidative stress in hepatic cells (Figure 3). In hepatocytes, disruption in mitochondrial membrane potential was observed, promoting cytochrome c release and apoptosis [56,57]. Additionally, efavirenz inhibits complex I of the ETC, stimulating ROS production and decreases in ATP [58]. In neuronal cells, the drug caused increased mitochondrial depolarisation, altered mitochondrial morphology, and ultimately led to mitophagy (clearance of damaged mitochondria) [59]. Mitochondrial dysfunction is considered to promote the progression of MetS by encouraging the occurrence of inflammation and oxidative stress. 

Efavirenz causes a decrease in ATP concentration and increases ROS production, which leads to an elevation in the lipid content of hepatic cells [58]. Furthermore, studies have shown that efavirenz acts as a pregnane X receptor agonist, which promotes the occurrence of dyslipidaemia and hypercholesterolemia [60].

**Figure 4:**
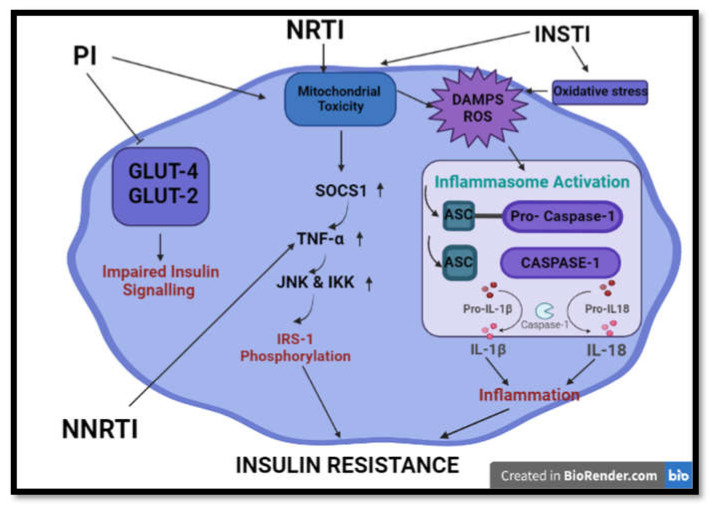
Summary of common processes implicated in HAART-induced insulin resistance and impaired insulin signalling. HAART induces insulin resistance and aberrations in insulin signalling via initiation of mitochondrial toxicity, oxidative stress, inflammation and inhibition of glucose transporters. (GLUT2/4- glucose transporter 2/4; IRS-1 -Insulin receptor 1; TNF-α—tumour nerosis factor alpha; SOCS1—suppressor of cytokine signaling 1; JNK—Jun N-terminal kinase; IKK-IκB kinase; ASC—Apoptosis-associated speck-like protein containing a CARD; IL-1β/18—interlukin 1 beta/ 18; DAMPS- damage activated molecular patterns).

In terms of insulin resistance, NNRTIs were found to reduce insulin sensitivity through its pro-inflammatory effects (Figure 4). Previous studies show that NNRTIs increase the expression of TNF-α, IL-6, and IL-1β, which results in decreased adiponectin concentrations. The reduction in the insulin sensitivity modulator allows for progression to insulin resistance, as previously discussed [61].

Research indicates that earlier NNRTIs were problematic and newer drugs in the class are seen to have greater resistance and fewer adversities [55].

## 8. Protease Inhibitors

Protease inhibitors are described as the most problematic ARVs in several studies. PIs work by inhibiting the function of HIV protease. In doing so, PIs prevent the cleavage of the precursor polyprotein needed to produce mature viral proteins necessary for infection [62]. Although this class of drugs is widely used in the treatment of HIV, the adverse outcomes following treatment are severe and are commonly associated with MetS.

Firstly, PIs are associated with mitochondrial dysfunction (Figure 3). The protease inhibitor (PI) ritonavir increases mtDNA copy number, increases ROS production, disrupts mitochondrial membrane potential, and interferes with respiration via its action on the ETC and OXPHOS [63]. Furthermore, it causes increases in oxidative stress and decreases in ATP synthesis [64]. Ritonavir promotes mitochondrial membrane potential changes, which initiates BAX translocation and cytochrome c release, allowing for apoptosis progression (Figure 3). Another PI, atazanavir, causes superoxide production in the mitochondria, depolarisation of the mitochondrial membrane, and apoptosis [65].

Furthermore, PIs are the most common ARVs associated with insulin resistance. PIs may induce insulin resistance through their effects on mitochondrial dysfunction, glucose transport, and inflammation induction. Upon mitochondrial damage or dysfunction, an increase in ROS is observed. This signals for an immune response via the action of the NALP3 inflammasome [66,67] (Figure 4). Inflammasomes are responsible for the clearance of pathogens and damaged cells. Furthermore, mitochondrial DAMPs such as mtDNA and DRP-1 may be released upon mitochondrial damage and are associated with inflammasome activation. The activation of inflammasomes promotes the release of caspase 1, which activates pro-inflammatory cytokines (IL-1β and IL-18). IL-1β was strongly linked with insulin resistance and impaired insulin secretion [68,69,70].

Previous in vitro studies showed that PIs cause the inhibition of the glucose 4 transporters (GLUT-4) [71]. This promotes impaired glucose tolerance and peripheral insulin resistance [72]. In liver cells, insulin signalling has been observed via action on GLUT-2. This transporter is responsible for hepatoportal glucose sensor function [71]. Furthermore, in therapeutic doses, PIs induce insulin resistance and beta pancreas cell damage in mice [72].

Prolonged exposure to PIs can affect insulin signalling and directly affect glucose uptake [73]. Saquinavir alters insulin signalling and IRS-1 phosphorylation. Indinavir activates the suppressor of cytokine signalling-1 (SOCS1), which causes an elevation in TNF-α levels [74]. TNF-α affects the IRS proteins that lead to insulin resistance. The cytokine induces activation of serine kinases such as JNK and IKK, which serine phosphorylates IRS-1. The increased concentration of phosphorylated IRS-1 inhibits the insulin receptor, thus causing insulin resistance [75].

In other studies, PIs are implicated in adipocyte differentiation inhibition via their action on the sterol regulatory element-binding protein-1 and peroxisome proliferator-activated receptor gamma (PPAR-γ). The inhibition in adipocyte differentiation alters lipid metabolism, leading to lipodystrophy and dyslipidaemia [76]. Furthermore, indinavir (PI) is associated with non-oxidative insulin-stimulated glucose disposal. This results in insulin resistance and dysregulation in lipid metabolism [77]. 

PI-mediated lipodystrophy is associated with irregular fat distribution and the accumulation of fat in the subcutaneous region [78,79]. The accumulation of fat causes an elevation in cholesterol and triglyceride levels, which leads to dyslipidaemia [80]. Furthermore, the use of PIs was associated with increased endoplasmic reticulum stress and the inhibition of proteasome action [79]. This results in autophagy inhibition, which is essential in regulating hepatic lipid metabolism and adipocyte lipid storage. The overall disruption leads to dyslipidaemia [81].

The plethora of evidence highlights the problems associated with PI usage and its implication in MetS progression. This has caused the PI class of drugs to be less favoured in recent years while newer drugs are being developed.

## 9. Nucleoside Reverse Transcriptase

NRTIs are one of the most common classes of ARVs. Drugs of this class are able to act as nucleoside analogues of HIV reverse transcriptase, thus terminating viral DNA synthesis in HIV [82]. Although NRTIs are popular in research, the plethora of studies indicate several adversities and side effects that allude to the progression of MetS.

The first observed effects of ARVs on the mitochondria were seen with NRTI use (Figure 3). NRTIs inhibit polymerase-γ, which has a role in mitochondrial DNA replication, thus compromising mitochondrial integrity [83]. Furthermore, NRTIs prevent ATP/ADP translocation. This causes impairment in respiration and ATP synthesis [84,85]. Studies show that decreases in mitochondrial DNA (mtDNA) content were linked to changes in respiration. The reduction in mtDNA initiates impairment in oxidative phosphorylation (OXPHOS) proteins and increases oxidative stress in mitochondria. This results in damage to mitochondrial proteins and lipids, further impairing mitochondrial function [86].

Furthermore, they cause alterations in nucleotide phosphorylation and directly affect mitochondrial respiration and ATP production [87,88]. Selected NRTIs reduce respiration and increase ROS via the inhibition of complex IV and I of the electron transport chain (ETC) [89,90,91] (Figure 3). This action has implications for mitochondrial dysfunction. ROS causes dysregulation of pathways leading to the release of damage-associated molecular patterns (DAMPS) and, thereafter, inflammatory activation [92].

In other instances, it was shown that tenofovir and tenofovir disoproxil fumarate (NRTI) were able to accumulate in renal cells, altering their viability and proliferation. This compromised mitochondrial function. The latter caused an increase in superoxide production and depletion of oxygen. Furthermore, a decrease in mitochondrial membrane potential was observed (Figure 3). The resultant effect was an increase in oxidative stress, which is hazardous to cells and implicated in T2DM and CVD pathogenesis [50]. Tenofovir disoproxil fumarate was found to decrease the function of ETC complexes I, II, IV, V, leading to reduced ATP production and proximal tubular damage in rat kidneys [93].

Mitochondrial dysfunction affects the occurrence of lipodystrophy. The generation of excessive ROS at sites of the ETC causes disruptions in adipocyte differentiation and induces apoptosis. At low concentrations, ROS may initiate lipogenesis and adipogenesis; however, at high concentrations, it may inhibit differentiation through the suppression of PPAR-γ [94]. The decrease in adipogenesis and induction of apoptosis leads to the development of lipodystrophy. NRTIs have been implicated in lipodystrophy development through its effects on oxidative stress and mitochondrial dysfunction [95,96].

## 10. Integrase Strand Transfer Inhibitors 

INSTIs are a relatively new class of ARVs that are gaining popularity due to their reduced side effects. Their novel mechanism of action includes preventing HIV integrase from incorporating pro-viral DNA into their host cell. This inhibits the HIV-catalysed strand transfer step. INSTIs are incredibly useful as this step has no known human analogue, increasing the specificity of the drug and reducing the toxicity [97]. However, research remains unable to fully explain the adversities associated with the usage of this class of drugs. Current research does implicate INSTIs in mitochondrial dysfunction, inflammation, and linkage to insulin resistance [63,98]. 

Dolutegravir and other INSTIs initiate increases in mtDNA number and ROS production [63]. Dolutegravir specifically is associated with decreased respiration of CD4^+^ T cells, excessive mitochondrial ROS production, and increased mitochondrial mass. This leads to mitochondrial dysfunction, slower cell proliferation rates, lower OXPHOS, and increased TNF-α responses [63].

More recently, INSTIs have been linked to insulin resistance, but literature remains limited (Figure 4). Dolutegravir was found to increase oxidative stress and promote lipid accumulation, favouring the onset of insulin resistance in adipose tissue and adipocytes. Furthermore, mitochondrial dysfunction was observed, which promotes insulin resistance. The results allowed the authors to infer that dolutegravir contributes to insulin resistance [98].

Considering the reduced side effects of the drugs and approval by WHO for usage in combinational treatment of HIV [99], research needs to be expanded to fully elucidate the mechanisms of toxicity of INSTIs.

## 11. ARVs and Epigenetic Modifications—Emerging Evidence

Studies regarding ARV usage and epigenetic modifications remain limited. Furthermore, studies that exist do not directly link MetS to ARV-induced epigenetic change, but rather provide evidence for strong possibilities and indirect links. This highlights the novelty of the concept and provides cause for extensive research to be undertaken in the subject area.

Unlike the epigenetic modifications induced by HIV, HAART-related epigenetic effects are mostly associated with altered micro-RNA (miRNA) expression. Interestingly, it was found that combinational ARV usage resulted in decreased expression of miR-106a and miR-140 and increased miR-192 levels. Lower levels of these miRNAs were seen to reduce CD4+ cell recovery, which is strongly connected to persistent inflammation and immune activation [100].

Recent epigenetic studies suggest that ritonavir (PI) alters miRNA expression. The increased levels of miR-28 were inversely proportional to the expression of the GLUT-4 transporter, implying that the drug alters glucose metabolism [101]. However, more studies are required to understand the epigenetic regulation of insulin resistance by PIs and establish a mechanism of action.

Besides altered miRNA expression, previous studies provide evidence that illustrates the role of NRTIs in causing the overexpression of DNA methyltransferase 1 (DNMT1). This was coupled with mtDNA hypermethylation in Hepatitis B infection [102]. Increases in DNMT1 are synonymous with decreased PPAR-γ expression and, thus, increased pro-inflammatory cytokine production in atherosclerosis patients’ blood monocytes [103]. Furthermore, increased DNMT1 in the adipocytes of obese mice correlates with hypermethylated adiponectin, which decreases the expression of vital energy homeostasis regulators [104]. 

Although pre-existing conditions may influence these changes, there are substantial data to encourage future epigenetic studies related to ARV usage and MetS. Furthermore, it is essential to note that HIV-infected individuals may have co-morbidities that favour the onset of epigenetic modifications

## 12. Conclusions and Recommendations 

This study provides an overview of the metabolic irregularities induced by HIV and HAART. We deduce that mitochondrial dysfunction is the most common underlying mechanism used by HIV and most ARVs to cause inflammation, insulin resistance, dyslipidaemia, and lipodystrophy. This information is vital as drugs used to treat HIV should be designed with less mitochondrial toxicity. Additionally, we found that most studies describe mechanisms of action following the use of ARVs individually. This is alarming as most ARV therapy is combinational, highlighting the need for mechanistic research into combinational ARV usage.

Considering the evidence, we can conclude that PIs are the most dangerous of ARVs due to their ability to initiate many toxicities leading to MetS. Furthermore, this paper addresses the urgency for more epigenetic experimentation to link HIV, HAART, and MetS. This novel approach may help fill current knowledge gaps and help prevent the side effects of the drugs. This is vital considering the global burden of HIV and deaths caused by HIV-related illness.

## Data Availability

Not applicable.

## References

[1] Saklayen M.G. (2018). The global epidemic of the metabolic syndrome. Curr. Hypertens. Rep..

[2] Masuku S.K.S., Tsoka-Gwegweni J., Sartorius B. (2019). HIV and antiretroviral therapy-induced metabolic syndrome in people living with HIV and its implications for care: A critical review. J. Diabetol..

[3] WHO Noncommunicable Diseases. https://www.who.int/news-room/fact-sheets/detail/noncommunicable-diseases.

[4] NHS Metabolic Syndrome. https://www.nhs.uk/conditions/metabolic-syndrome/.

[5] Prasun P. (2021). Mitochondrial dysfunction in metabolic syndrome. Biochim. Biophys. Acta (BBA) Mol. Basis Dis..

[6] Esser N., Legrand-Poels S., Piette J., Scheen A.J., Paquot N. (2014). Inflammation as a link between obesity, metabolic syndrome and type 2 diabetes. Diabetes Res. Clin. Pract..

[7] Vona R., Gambardella L., Cittadini C., Straface E., Pietraforte D. (2019). Biomarkers of oxidative stress in metabolic syndrome and associated diseases. Oxidative Med. Cell. Longev..

[8] Carson C., Lawson H.A. (2018). Epigenetics of metabolic syndrome. Physiol. Genom..

[9] Todowede O.O., Mianda S.Z., Sartorius B. (2019). Prevalence of metabolic syndrome among HIV-positive and HIV-negative populations in sub-Saharan Africa—a systematic review and meta-analysis. Syst. Rev..

[10] UNAIDS Global HIV & AIDS Statistics—2020 Fact Sheet. https://www.unaids.org/en/resources/fact-sheet.

[11] Stats-SA 2020 Mid-Year Population Estimates. http://www.statssa.gov.za/?p=13453.

[12] WHO HIV/AIDS. https://www.who.int/news-room/fact-sheets/detail/hiv-aids.

[13] Chhoun P., Tuot S., Harries A.D., Kyaw N.T.T., Pal K., Mun P., Brody C., Mburu G., Yi S. (2017). High prevalence of non-communicable diseases and associated risk factors amongst adults living with HIV in Cambodia. PLoS ONE.

[14] Hyle E.P., Naidoo K., Su A.E., El-Sadr W.M., Freedberg K.A. (2014). HIV, tuberculosis, and non-communicable diseases: What is known about the costs, effects, and cost-effectiveness of integrated care?. J. Acquir. Immune Defic. Syndr..

[15] Ergin H.E., Inga E.E., Maung T.Z., Javed M., Khan S. (2020). HIV, antiretroviral therapy and metabolic alterations: A review. Cureus.

[16] Jaggers J.R., Prasad V.K., Dudgeon W.D., Blair S.N., Sui X., Burgess S., Hand G.A. (2014). Associations between physical activity and sedentary time on components of metabolic syndrome among adults with HIV. AIDS Care.

[17] Nguyen K., Peer N., Mills E.J., Kengne A.P. (2016). A meta-analysis of the metabolic syndrome prevalence in the global HIV-infected population. PLoS ONE.

[18] Syed F.F., Sani M.U. (2013). Recent advances in HIV-associated cardiovascular diseases in Africa. Heart.

[19] Nguyen K., Peer N., De Villiers A., Mukasa B., Matsha T.E., Mills E.J., Kengne A.P. (2017). Metabolic syndrome in people living with human immunodeficiency virus: An assessment of the prevalence and the agreement between diagnostic criteria. Int. J. Endocrinol..

[20] Motala A.A., Esterhuizen T., Pirie F.J., Omar M.A. (2011). The prevalence of metabolic syndrome and determination of the optimal waist circumference cutoff points in a rural South African community. Diabetes Care.

[21] Erasmus R.T., Soita D.J., Hassan M.S., Blanco-Blanco E., Vergotine Z., Kengne A.P., Matsha T.E. (2012). High prevalence of diabetes mellitus and metabolic syndrome in a South African coloured population: Baseline data of a study in Bellville, Cape Town. S. Afr. Med. J..

[22] Nasi M., De Biasi S., Gibellini L., Bianchini E., Pecorini S., Bacca V., Guaraldi G., Mussini C., Pinti M., Cossarizza A. (2017). Ageing and inflammation in patients with HIV infection. Clin. Exp. Immunol..

[23] Merrill J.E., Koyanagi Y., Chen I. (1989). Interleukin-1 and tumor necrosis factor alpha can be induced from mononuclear phagocytes by human immunodeficiency virus type 1 binding to the CD4 receptor. J. Virol..

[24] Rieckmann P., Poli G., Fox C.H., Kehrl J.H., Fauci A.S. (1991). Recombinant gp120 specifically enhances tumor necrosis factor-alpha production and Ig secretion in B lymphocytes from HIV-infected individuals but not from seronegative donors. J. Immunol..

[25] Lee C., Liu Q.H., Tomkowicz B., Yi Y., Freedman B.D., Collman R.G. (2003). Macrophage activation through CCR5-and CXCR4-mediated gp120-elicited signaling pathways. J. Leukoc. Biol..

[26] Wang J.-K., Kiyokawa E., Verdin E., Trono D. (2000). The Nef protein of HIV-1 associates with rafts and primes T cells for activation. Proc. Natl. Acad. Sci. USA.

[27] Simmons A., Aluvihare V., McMichael A. (2001). Nef triggers a transcriptional program in T cells imitating single-signal T cell activation and inducing HIV virulence mediators. Immunity.

[28] Swingler S., Mann A., Jacque J.-M., Brichacek B., Sasseville V., Williams K., Lackner A., Janoff E., Wang R., Fisher D. (1999). HIV-1 Nef mediates lymphocyte chemotaxis and activation by infected macrophages. Nat. Med..

[29] Brenchley J.M., Price D.A., Schacker T.W., Asher T.E., Silvestri G., Rao S., Kazzaz Z., Bornstein E., Lambotte O., Altmann D. (2006). Microbial translocation is a cause of systemic immune activation in chronic HIV infection. Nat. Med..

[30] Appay V., Sauce D. (2008). Immune activation and inflammation in HIV-1 infection: Causes and consequences. J. Pathol. J. Pathol. Soc. Great Br. Irel..

[31] Guo H., Callaway J.B., Ting J.P. (2015). Inflammasomes: Mechanism of action, role in disease, and therapeutics. Nat. Med..

[32] Mullis C., Swartz T.H. (2020). NLRP3 Inflammasome signaling as a link between HIV-1 infection and atherosclerotic cardiovascular disease. Front. Cardiovasc. Med..

[33] Yudkin J. (2003). Adipose tissue, insulin action and vascular disease: Inflammatory signals. Int. J. Obes..

[34] Caballero A.E. (2004). Endothelial dysfunction, inflammation, and insulin resistance: A focus on subjects at risk for type 2 diabetes. Curr. Diabetes Rep..

[35] Matsuzawa Y., Funahashi T., Kihara S., Shimomura I. (2004). Adiponectin and metabolic syndrome. Arterioscler. Thromb. Vasc. Biol..

[36] Ziemke F., Mantzoros C.S. (2010). Adiponectin in insulin resistance: Lessons from translational research. Am. J. Clin. Nutr..

[37] Jensen T.E., Sylow L., Rose A.J., Madsen A.B., Angin Y., Maarbjerg S.J., Richter E.A. (2014). Contraction-stimulated glucose transport in muscle is controlled by AMPK and mechanical stress but not sarcoplasmatic reticulum Ca2+ release. Mol. Metab..

[38] Kim Y., Park C.W. (2019). Mechanisms of adiponectin action: Implication of adiponectin receptor agonism in diabetic kidney disease. Int. J. Mol. Sci..

[39] Roda R.H., Hoke A. (2019). Mitochondrial dysfunction in HIV-induced peripheral neuropathy. Int. Rev. Neurobiol..

[40] Grunfeld C., Pang M., Doerrler W., Shigenaga J., Jensen P., Feingold K. (1992). Lipids, lipoproteins, triglyceride clearance, and cytokines in human immunodeficiency virus infection and the acquired immunodeficiency syndrome. J. Clin. Endocrinol. Metab..

[41] Haugaard S.B., Andersen O., Pedersen S.B., Dela F., Fenger M., Richelsen B., Madsbad S., Iversen J. (2006). Tumor necrosis factor α is associated with insulin-mediated suppression of free fatty acids and net lipid oxidation in HIV-infected patients with lipodystrophy. Metabolism.

[42] Guilherme A., Virbasius J.V., Puri V., Czech M.P. (2008). Adipocyte dysfunctions linking obesity to insulin resistance and type 2 diabetes. Nat. Rev. Mol. Cell Biol..

[43] Karpe F., Dickmann J.R., Frayn K.N. (2011). Fatty acids, obesity, and insulin resistance: Time for a reevaluation. Diabetes.

[44] Maseko T.S., Masuku S.K. (2017). The effect of HIV and ART on the development of hypertension and type 2 diabetes mellitus. J. Diabetes Metab..

[45] Espíndola M.S., Soares L.S., Galvão-Lima L.J., Zambuzi F.A., Cacemiro M.C., Brauer V.S., Marzocchi-Machado C.M., de Souza Gomes M., Amaral L.R., Martins-Filho O.A. (2018). Epigenetic alterations are associated with monocyte immune dysfunctions in HIV-1 infection. Sci. Rep..

[46] Sundermann E.E., Hussain M.A., Moore D.J., Horvath S., Lin D.T., Kobor M.S., Levine A. (2019). Inflammation-related genes are associated with epigenetic aging in HIV. J. Neurovirol..

[47] Bogoi R.N., de Pablo A., Valencia E., Martín-Carbonero L., Moreno V., Vilchez-Rueda H.H., Asensi V., Rodriguez R., Toledano V., Rodés B. (2018). Expression profiling of chromatin-modifying enzymes and global DNA methylation in CD4+ T cells from patients with chronic HIV infection at different HIV control and progression states. Clin. Epigenetics.

[48] Nakayama-Hosoya K., Ishida T., Youngblood B., Nakamura H., Hosoya N., Koga M., Koibuchi T., Iwamoto A., Kawana-Tachikawa A. (2015). Epigenetic repression of interleukin 2 expression in senescent CD4+ T cells during chronic HIV type 1 infection. J. Infect. Dis..

[49] Youngblood B., Oestreich K.J., Ha S.-J., Duraiswamy J., Akondy R.S., West E.E., Wei Z., Lu P., Austin J.W., Riley J.L. (2011). Chronic virus infection enforces demethylation of the locus that encodes PD-1 in antigen-specific CD8+ T cells. Immunity.

[50] Milián L., Peris J.E., Gandía P., Andújar I., Pallardó L., Górriz J.L., Blas-García A. (2017). Tenofovir-induced toxicity in renal proximal tubular epithelial cells: Involvement of mitochondria. Aids.

[51] Paula A.A., Falcão M.C., Pacheco A.G. (2013). Metabolic syndrome in HIV-infected individuals: Underlying mechanisms and epidemiological aspects. AIDS Res. Ther..

[52] Dau B., Holodniy M. (2008). The relationship between HIV infection and cardiovascular disease. Curr. Cardiol. Rev..

[53] Muyanja D., Muzoora C., Muyingo A., Muyindike W., Siedner M.J. (2016). High prevalence of metabolic syndrome and cardiovascular disease risk among people with HIV on stable ART in southwestern Uganda. AIDS Patient Care STDs.

[54] Obirikorang C., Quaye L., Osei-Yeboah J., Odame E.A., Asare I. (2016). Prevalence of metabolic syndrome among HIV-infected patients in Ghana: A cross-sectional study. Niger. Med. J. J. Niger. Med. Assoc..

[55] De Béthune M.-P. (2010). Non-nucleoside reverse transcriptase inhibitors (NNRTIs), their discovery, development, and use in the treatment of HIV-1 infection: A review of the last 20 years (1989–2009). Antivir. Res..

[56] Jamaluddin M.S., Lin P.H., Yao Q., Chen C. (2010). Non-nucleoside reverse transcriptase inhibitor efavirenz increases monolayer permeability of human coronary artery endothelial cells. Atherosclerosis.

[57] Ganta K.K., Mandal A., Chaubey B. (2017). Depolarization of mitochondrial membrane potential is the initial event in non-nucleoside reverse transcriptase inhibitor efavirenz induced cytotoxicity. Cell Biol. Toxicol..

[58] Blas-García A., Apostolova N., Ballesteros D., Monleon D., Morales J.M., Rocha M., Victor V.M., Esplugues J.V. (2010). Inhibition of mitochondrial function by efavirenz increases lipid content in hepatic cells. Hepatology.

[59] Purnell P.R., Fox H.S. (2014). Efavirenz induces neuronal autophagy and mitochondrial alterations. J. Pharmacol. Exp. Ther..

[60] Gwag T., Meng Z., Sui Y., Helsley R.N., Park S.-H., Wang S., Greenberg R.N., Zhou C. (2019). Non-nucleoside reverse transcriptase inhibitor efavirenz activates PXR to induce hypercholesterolemia and hepatic steatosis. J. Hepatol..

[61] Lagathu C., Kim M., Maachi M., Vigouroux C., Cervera P., Capeau J., Caron M., Bastard J.-P. (2005). HIV antiretroviral treatment alters adipokine expression and insulin sensitivity of adipose tissue in vitro and in vivo. Biochimie.

[62] De Clercq E. (2013). The nucleoside reverse transcriptase inhibitors, nonnucleoside reverse transcriptase inhibitors, and protease inhibitors in the treatment of HIV infections (AIDS). Adv. Pharmacol..

[63] Korencak M., Byrne M., Richter E., Schultz B.T., Juszczak P., Ake J.A., Ganesan A., Okulicz J.F., Robb M.L., de Los Reyes B. (2019). Effect of HIV infection and antiretroviral therapy on immune cellular functions. JCI Insight.

[64] Wang X., Mu H., Chai H., Liao D., Yao Q., Chen C. (2007). Human immunodeficiency virus protease inhibitor ritonavir inhibits cholesterol efflux from human macrophage-derived foam cells. Am. J. Pathol..

[65] Gibellini L., De Biasi S., Pinti M., Nasi M., Riccio M., Carnevale G., Cavallini G.M., de Oyanguren F.J.S., O’Connor J.E., Mussini C. (2012). The protease inhibitor atazanavir triggers autophagy and mitophagy in human preadipocytes. Aids.

[66] Nakahira K., Haspel J.A., Rathinam V.A., Lee S.-J., Dolinay T., Lam H.C., Englert J.A., Rabinovitch M., Cernadas M., Kim H.P. (2011). Autophagy proteins regulate innate immune responses by inhibiting the release of mitochondrial DNA mediated by the NALP3 inflammasome. Nat. Immunol..

[67] Shimada K., Crother T.R., Karlin J., Dagvadorj J., Chiba N., Chen S., Ramanujan V.K., Wolf A.J., Vergnes L., Ojcius D.M. (2012). Oxidized mitochondrial DNA activates the NLRP3 inflammasome during apoptosis. Immunity.

[68] Stienstra R., Joosten L.A., Koenen T., Van Tits B., Van Diepen J.A., Van Den Berg S.A., Rensen P.C., Voshol P.J., Fantuzzi G., Hijmans A. (2010). The inflammasome-mediated caspase-1 activation controls adipocyte differentiation and insulin sensitivity. Cell Metab..

[69] Vandanmagsar B., Youm Y.-H., Ravussin A., Galgani J.E., Stadler K., Mynatt R.L., Ravussin E., Stephens J.M., Dixit V.D. (2011). The NLRP3 inflammasome instigates obesity-induced inflammation and insulin resistance. Nat. Med..

[70] Wen H., Gris D., Lei Y., Jha S., Zhang L., Huang M.T.-H., Brickey W.J., Ting J.P. (2011). Fatty acid–induced NLRP3-ASC inflammasome activation interferes with insulin signaling. Nat. Immunol..

[71] Murata H., Hruz P.W., Mueckler M. (2000). The mechanism of insulin resistance caused by HIV protease inhibitor therapy. J. Biol. Chem..

[72] Koster J.C., Remedi M.S., Qiu H., Nichols C.G., Hruz P.W. (2003). HIV protease inhibitors acutely impair glucose-stimulated insulin release. Diabetes.

[73] Ben-Romano R., Rudich A., Török D., Vanounou S., Riesenberg K., Schlaeffer F., Klip A., Bashan N. (2003). Agent and cell-type specificity in the induction of insulin resistance by HIV protease inhibitors. AIDS.

[74] Carper M.J., Cade W.T., Cam M., Zhang S., Shalev A., Yarasheski K.E., Ramanadham S. (2008). HIV-protease inhibitors induce expression of suppressor of cytokine signaling-1 in insulin-sensitive tissues and promote insulin resistance and type 2 diabetes mellitus. Am. J. Physiol. Endocrinol. Metab..

[75] Ismail W., King J., Pillay T.S. (2009). Insulin resistance induced by antiretroviral drugs: Current understanding of molecular mechanisms. J. Endocrinol. Metab. Diabetes S. Afr..

[76] Geleziunas R., Xu W., Takeda K., Ichijo H., Greene W.C. (2001). HIV-1 Nef inhibits ASK1-dependent death signalling providing a potential mechanism for protecting the infected host cell. Nature.

[77] Noor M.A., Seneviratne T., Aweeka F.T., Lo J.C., Schwarz J.-M., Mulligan K., Schambelan M., Grunfeld C. (2002). Indinavir acutely inhibits insulin-stimulated glucose disposal in humans: A randomized, placebo-controlled study. AIDS.

[78] Anuurad E., Bremer A., Berglund L. (2010). HIV protease inhibitors and obesity. Curr. Opin. Endocrinol. Diabetes Obes..

[79] Zha B.S., Wan X., Zhang X., Zha W., Zhou J., Wabitsch M., Wang G., Lyall V., Hylemon P.B., Zhou H. (2013). HIV protease inhibitors disrupt lipid metabolism by activating endoplasmic reticulum stress and inhibiting autophagy activity in adipocytes. PLoS ONE.

[80] Feingold K.R. (2020). Obesity and dyslipidemia. Endotext.

[81] Czaja M.J. (2010). Autophagy in health and disease. 2. Regulation of lipid metabolism and storage by autophagy: Pathophysiological implications. Am. J. Physiol. Cell Physiol..

[82] Holec A.D., Mandal S., Prathipati P.K., Destache C.J. (2017). Nucleotide reverse transcriptase inhibitors: A thorough review, present status and future perspective as HIV therapeutics. Curr. HIV Res..

[83] Schank M., Zhao J., Moorman J.P., Yao Z.Q. (2021). The Impact of HIV-and ART-Induced Mitochondrial Dysfunction in Cellular Senescence and Aging. Cells.

[84] Barile M., Valenti D., Hobbs G.A., Abruzzese M.F., Keilbaugh S.A., Passarella S., Quagliariello E., Simpson M.V. (1994). Mechanisms of toxicity of 3′-azido-3′-deoxythymidine: Its interaction with adenylate kinase. Biochem. Pharmacol..

[85] Barile M., Valenti D., Passarella S., Quagliariello E. (1997). 3′-Azido-3′-deoxythymidine uptake into isolated rat liver mitochondria and impairment of ADP/ATP translocator. Biochem. Pharmacol..

[86] Lewis W., Day B.J., Copeland W.C. (2003). Mitochondrial toxicity of NRTI antiviral drugs: An integrated cellular perspective. Nat. Rev. Drug Discov..

[87] Höschele D. (2006). Cell culture models for the investigation of NRTI-induced mitochondrial toxicity: Relevance for the prediction of clinical toxicity. Toxicol. Vitr..

[88] Mallon P.W., Miller J., Cooper D.A., Carr A. (2003). Prospective evaluation of the effects of antiretroviral therapy on body composition in HIV-1-infected men starting therapy. AIDS.

[89] Lund K.C., Wallace K.B. (2008). Adenosine 3′, 5′-cyclic monophosphate (cAMP)-dependent phosphoregulation of mitochondrial complex I is inhibited by nucleoside reverse transcriptase inhibitors. Toxicol. Appl. Pharmacol..

[90] Maagaard A., Kvale D. (2009). Long term adverse effects related to nucleoside reverse transcriptase inhibitors: Clinical impact of mitochondrial toxicity. Scand. J. Infect. Dis..

[91] Cote H.C., Brumme Z.L., Craib K.J., Alexander C.S., Wynhoven B., Ting L., Wong H., Harris M., Harrigan P.R., O’Shaughnessy M.V. (2002). Changes in mitochondrial DNA as a marker of nucleoside toxicity in HIV-infected patients. N. Engl. J. Med..

[92] Li M., Mislak A.C., Foli Y., Agbosu E., Bose V., Bhandari S., Szymanski M.R., Shumate C.K., Yin Y.W., Anderson K.S. (2016). The DNA polymerase gamma R953C mutant is associated with antiretroviral therapy-induced mitochondrial toxicity. Antimicrob. Agents Chemother..

[93] Ramamoorthy H., Abraham P., Isaac B. (2014). Mitochondrial dysfunction and electron transport chain complex defect in a rat model of tenofovir disoproxil fumarate nephrotoxicity. J. Biochem. Mol. Toxicol..

[94] Furukawa S., Fujita T., Shimabukuro M., Iwaki M., Yamada Y., Nakajima Y., Nakayama O., Makishima M., Matsuda M., Shimomura I. (2004). Increased oxidative stress in obesity and its impact on metabolic syndrome. J. Clin. Investig..

[95] Gerschenson M., Kim C., Berzins B., Taiwo B., Libutti D.E., Choi J., Chen D., Weinstein J., Shore J., Da Silva B. (2009). Mitochondrial function, morphology and metabolic parameters improve after switching from stavudine to a tenofovir-containing regimen. J. Antimicrob. Chemother..

[96] Boothby M., McGee K.C., Tomlinson J.W., Gathercole L.L., McTernan P.G., Shojaee-Moradie F., Umpleby A.M., Nightingale P., Shahmanesh M. (2009). Adipocyte differentiation, mitochondrial gene expression and fat distribution: Differences between zidovudine and tenofovir after 6 months. Antivir. Ther..

[97] Dow D.E., Bartlett J.A. (2014). Dolutegravir, the second-generation of integrase strand transfer inhibitors (INSTIs) for the treatment of HIV. Infect. Dis. Ther..

[98] Gorwood J., Bourgeois C., Pourcher V., Pourcher G., Charlotte F., Mantecon M., Rose C., Morichon R., Atlan M., Le Grand R. (2020). The integrase inhibitors dolutegravir and raltegravir exert proadipogenic and profibrotic effects and induce insulin resistance in human/simian adipose tissue and human adipocytes. Clin. Infect. Dis..

[99] WHO Updated Recommendations on First-Line and Second-Line Antiretroviral Regimens and Post-Exposure Prophylaxis and Recommendations on Early Infant Diagnosis of HIV. https://www.who.int/publications/i/item/WHO-CDS-HIV-18.51.

[100] Hernández-Walias F., Ruiz-de-León M.J., Rosado-Sánchez I., Vázquez E., Leal M., Moreno S., Vidal F., Blanco J., Pacheco Y.M., Vallejo A. (2020). New signatures of poor CD4 cell recovery after suppressive antiretroviral therapy in HIV-1-infected individuals: Involvement of miR-192, IL-6, sCD14 and miR-144. Sci. Rep..

[101] Bresciani E., Saletti C., Squillace N., Rizzi L., Molteni L., Meanti R., Omeljaniuk R.J., Biagini G., Gori A., Locatelli V. (2019). miRNA-218 targets lipin-1 and glucose transporter type 4 genes in 3T3-L1 cells treated with lopinavir/ritonavir. Front. Pharmacol..

[102] Madeddu G., Ortu S., Garrucciu G., Maida I., Melis M., Muredda A.A., Mura M.S., Babudieri S. (2017). DNMT1 modulation in chronic hepatitis B patients and hypothetic influence on mitochondrial DNA methylation status during long-term nucleo (t) side analogs therapy. J. Med. Virol..

[103] Yu J., Qiu Y., Yang J., Bian S., Chen G., Deng M., Kang H., Huang L. (2016). DNMT1-PPARγ pathway in macrophages regulates chronic inflammation and atherosclerosis development in mice. Sci. Rep..

[104] Kim A.Y., Park Y.J., Pan X., Shin K.C., Kwak S.-H., Bassas A.F., Sallam R.M., Park K.S., Alfadda A.A., Xu A. (2015). Obesity-induced DNA hypermethylation of the adiponectin gene mediates insulin resistance. Nat. Commun..

